# Thyroid-stimulating hormone decreases the risk of osteoporosis by regulating osteoblast proliferation and differentiation

**DOI:** 10.1186/s12902-021-00715-8

**Published:** 2021-03-16

**Authors:** Tuo Deng, Wenwen Zhang, Yanling Zhang, Mengqi Zhang, Zhikun Huan, Chunxiao Yu, Xiujuan Zhang, Yan Wang, Jin Xu

**Affiliations:** 1 Department of Endocrinology, Shandong Provincial Hospital, Cheeloo College of Medicine, Shandong University, Jinan, 250021 Shandong China; 2 Shandong Provincial Key Laboratory of Endocrinology and Lipid Metabolism, Jinan, 250021 Shandong China; 3 Shandong Institute of Endocrine and Metabolic Disease, Jinan, 250021 Shandong China; 4 Department of Endocrinology, Shandong Provincial Hospital Affiliated to Shandong First Medical University, Jinan, 250021 Shandong China; 5 Department of Anesthesiology, Shandong Provincial Hospital, Cheeloo College of Medicine, Shandong University, Jinan, 250021 Shandong China

**Keywords:** Osteoporosis, TSH, BMD, Osteoblast

## Abstract

**Background:**

As the incidence of secretory osteoporosis has increased, bone loss, osteoporosis and their relationships with thyroid-stimulating hormone (TSH) have received increased attention. In this study, the role of TSH in bone metabolism and its possible underlying mechanisms were investigated.

**Methods:**

We analyzed the serum levels of free triiodothyronine (FT3), free thyroxine (FT4), and TSH and the bone mineral density (BMD) levels of 114 men with normal thyroid function. In addition, osteoblasts from rat calvarial samples were treated with different doses of TSH for different lengths of time. The related gene and protein expression levels were investigated.

**Results:**

A comparison of the BMD between the high-level and low-level serum TSH groups showed that the TSH serum concentration was positively correlated with BMD. TSH at concentrations of 10 mU/mL and 100 mU/mL significantly increased the mRNA levels of ALP, COI1 and Runx2 compared with those of the control (*P* < 0.05, *P* < 0.01). Bone morphogenetic protein (BMP)2 activity was enhanced with both increased TSH concentration and increased time. The protein levels of Runx2 and osterix were increased in a dose-dependent manner.

**Conclusions:**

The circulating concentrations of TSH and BMD were positively correlated with normal thyroid function in males. TSH promoted osteoblast proliferation and differentiation in rat primary osteoblasts.

**Supplementary Information:**

The online version contains supplementary material available at 10.1186/s12902-021-00715-8.

## Background

Osteoporosis is the seventh most common disease in the world. Due to the increase in life expectancy in an aging society, it has become a serious public health problem. This situation can lead to fractures, which will seriously affect the individual’s physical and mental health and quality of life. In recent years, due to the increasing incidence of secretory osteoporosis, bone loss, osteoporosis and their relationship with thyroid-stimulating hormone (TSH) and thyroid-stimulating hormone receptor (TSHR) have attracted increasing attention.

Homeostasis requires a balance between bone resorption and bone formation. Osteoblasts and osteoclasts continue to remodel bone [1–3] unless the balance is disrupted, which may lead to osteoporosis and bone sclerosis [4].

It is known that hyperthyroidism is associated with significant bone loss [5]. Subclinical hyperthyroidism (SH) means that serum free triiodothyronine (FT3) and free thyroxine (FT4) levels are normal, but TSH levels are low. Although patients with this diagnosis usually have no symptoms, there is sufficient evidence that osteoporosis is related to bone metabolism [6]. The main reasons for exogenous SH are improper T4 replacement dose and TSH inhibitory L-T4 in the treatment of benign thyroid nodules and thyroid cancer. The inhibition of TSH induced by L-T4 treatment can accelerate bone metabolism [7]. Existing data also suggest that long-term use of the T4 inhibitor TSH can reduce bone density and may increase the risk of fracture [8]. In the previous study, based on predetermined inclusion and exclusion criteria, 275,086 participants were included in 12 cohort studies and followed up for 3 months to 13 years. The results showed that SH had no effect on lumbar spine bone mineral density, whether it was male or female. However, female femoral neck bone mineral density was significantly reduced, while males did not. In addition, there was a significant increase in hip fractures in women and men with SH [9]. Studies have shown that after L-T4 treatment, serum TSH levels in postmenopausal women decrease, and the annual bone loss rate of postmenopausal women after 9.9 years is significantly higher than 0.91% in the control group [10]. However, three longitudinal studies evaluated changes in bone mineral density (BMD) and bone turnover in postmenopausal women with differentiated thyroid cancer (DTC) after L-T4 treatment [11–13], with conflicting results. A cross-sectional study showed that long-term use of L-T4 suppression therapy during peak bone mass may not have any significant adverse effects on bone density or the microstructure of bone density [14]. Therefore, the relationship between SH and osteoporosis is unclear.

TSH is a glycoprotein hormone produced by thyroid trophoblast cells of the anterior pituitary gland that plays an important role in regulating the development and function of the thyroid. In thyroid tissue, the effect of TSH is mediated by TSHR, which is a member of the seven transmembrane helix g protein-coupled receptor family. Osteoporosis is related to thyroid dysfunction and is traditionally considered to be a secondary result of changes in thyroid function [15]. However, in subsequent studies, the expression of TSHR mRNA and protein in normal osteoblasts was observed, suggesting that TSH may directly affect bone formation [16].

The expression of TSHR mRNA and protein in osteoblasts and osteoclasts was observed [17–20]. However, TSHRa and TSHRb were not detected in primary osteoblasts and osteoclasts [18], indicating that TSH has no paracrine effect on these cells [21]. Our initial mouse tissue morphology experiments showed that compared with Tshr+/+ mice, Tshr−/− and Tshr+/− mice had significantly reduced bone trabecular volume, bone-like surface, bone-like thickness, and osteoblast surface. The surface of osteoclasts increased significantly. In addition, TSH inhibits the differentiation of osteoclasts in vitro, which is manifested by a decrease in the number of tartrate-resistant acid phosphatase (TRAP)-positive cells and a decrease in the levels of differentiation markers such as TRAP, matrix metalloproteinase 9, proteinase K, etc. in RAW264.7 cells [4]. Our results confirm that TSH at least partially increases bone volume, improves bone microstructure, and improves bone strength by inhibiting the formation of osteoclasts.

The effect of TSH on osteoblasts is controversial. The model system used by Baliram includes mouse embryonic stem cells (ES), which are induced to form mature mineralized osteoblasts. TSH promotes the differentiation of osteoblasts mainly by activating protein kinase C and upregulating Fri4 and Wnt5a, the intermediate products of the atypical Wnt signaling pathway [22]. Sampath et al. showed that TSH stimulates osteoblast differentiation [19]. However, Tsai et al. previously only observed low levels of TSHR expression, TSH binding and cAMP activation in human osteoblasts and speculated that TSH is unlikely to play a physiological role in the blood-brain barrier of osteoblasts [23]. In contrast, TSH inhibits osteoblast differentiation and the expression of type I collagen in a Runx2- and osterix-independent manner by reducing the activity of Wnt and VEGF signaling pathways. TSH inhibits osteoblasts and reduces the expression of type I collagen, bone sialoprotein and osteocalcin. The inhibition of LDL receptor-related protein 5 (LRP5) mRNA expression indicates that these effects are mediated by the Wnt signaling pathway [17].

Therefore, although TSHR is expressed in osteoblasts, recent studies have yielded conflicting results, suggesting that TSH may enhance, inhibit, or not affect osteoblast differentiation and the function of [21].

In this report, a cross-sectional study of 114 Chinese men with normal thyroid function was conducted to determine serum TSH, FT3, and FT4 levels and BMD. In addition, the effects of TSH on the proliferation and differentiation of osteoblasts during the primary development of rat osteoblasts were also analyzed.

## Methods

### Subjects

The research subjects were selected by the Jinan City Health Organization from October to November 2009. A total of 731 men were recruited. The exclusion criteria included participants with thyroid disease, chronic diseases, liver disease, kidney disease, or other endocrine diseases and taking thyroid hormones, glucocorticoids, bisphosphonates, calcitonin, calcium, or active vitamin D analogs. In total, 114 men were included in the current analysis. All study participants must complete a consent form. This study was approved by the Ethics Committee of Shandong Provincial Hospital.

### Sample collection

The questionnaire survey provided information about each participant, including general information such as name, date of birth, address, ID number, telephone number, ethnicity, education level, occupation, income, health insurance status, iodine and food intake, smoking and drinking history, family and personal history of diseases and treatment methods. Height and weight were measured, and body mass index (BMI) was calculated with the following formula: BMI = weight (kg)/(height (m)^2). Fasting blood samples were taken for serum TSH, FT3 and FT4 analysis.

### Analysis of blood samples

Serum TSH, FT3 and FT4 concentrations were measured in the clinical laboratory of Shandong Provincial Hospital affiliated with Shandong University by electrochemiluminescence immunoassay (CobasE601, Roche, Basel, Switzerland). The normal reference range of TSH is 0.55 ~ 4.78 mU/L, and the normal reference ranges of FT3 and FT4 are 3.5 ~ 6.5 pmol/L and 11.5 ~ 22.7 pmol/L, respectively.

### Bone mineral density measurement

A well-trained technician used a dual-energy X-ray bone densitometer (DXA) sector bone densitometer (Japan Osteosys Co., Ltd., EXA-3000) to measure the bone mineral density in the low-median area of ​​the left forearm. According to the diagnostic criteria of the World Health Organization (WHO), osteoporosis is defined as BMD t score < 2.5, and BMD t score>-1 is normal. BMD T-scores between − 2.5 and − 1 are defined as osteopenia [24].

### Primary culture of osteoblasts

For primary culture of osteoblasts, a slightly modified sequential digestion method was used to isolate osteoblasts from the skulls of newborn rat pups. Briefly, we isolated heads from 8 newborn (1–3 days old) rat pups. After the sutures and adherent mesenchymal tissue were removed, the skull was placed in a water bath containing 0.25% trypsin and digested at 37 °C and 200 rpm for 30 min. The precipitate was collected, and a second digestion was performed in a test tube containing 0.1% type II collagenase in a water batch at a temperature of 37 °C at a rotation speed of 200 rpm. The cells were extracted from the supernatant, washed twice with PBS, resuspended in DMEM/F12 medium containing 10% fetal bovine serum, 1% penicillin/streptomycin and 1% glutamine solution, and transferred to a petri dish for incubation at 37 °C in 5% CO_2_ in an incubator.

### Osteoblast phenotype and function identification

Third-generation osteoblasts were cultured at a density of 5*10^4^ cells/ml in a 12-well plate. Hematoxylin-eosin staining was used to observe the typical morphological characteristics of osteoblasts. Alkaline phosphatase (ALP) staining was performed using an ALP kit (GENMED, China) according to the manufacturer’s protocol. Alkaline phosphatase-positive cells with 3 or more nuclei were defined as osteoblasts. Skull-derived osteoblasts were inoculated at a density of 5*10^4^ cells/mL and cultured in DMEM/F12 supplemented with 10% fetal bovine serum, 1% penicillin/streptomycin and 1% glutamine for 16 days. Alizarin red staining was used to detect osteoblast mineralization. For Alizarin red staining, the cells were also plated and fixed, stained with 2% Alizarin red for 5 min, washed several times with PBS, and air-dried at room temperature. Finally, the cells were observed under a microscope.

### Osteoblast growth curve

Confluent P3-generation osteoblasts were used to prepare a single cell suspension, and the cell concentration was adjusted to 2.5*10^4^ cells/ml. Then, 200 μL of cells/well was inoculated onto a 96-well plate. After inoculation, 6 cell samples were selected for the MTT colorimetric test every 24 h for 7 days. The specific steps were as follows: 20 μL of 5 mg/mL MTT solution was added to each well, and the cells were incubated at 37 °C. After 4 h, the culture medium was discarded, 150 μL of dimethyl sulfoxide (DMSO) was added, and the dark blue formazan crystals were solubilized at 37 °C for 10 min. The absorbance was recorded at 490 nm with an automatic microplate reader. The growth curve was generated with time on the x-axis and the OD value on the y-axis.

### Cell proliferation

The MTT method was used to measure the proliferation of TSH-treated cells. Skull osteoblasts were suspended in DMEM/F12 medium and plated at a density of 2.5*10^4^ cells/well in a 96-well plate until the cells were close to confluence. The cells were serum-starved for 2 h, after which TSH was added to the appropriate wells at different concentrations (0, 1, 10, 100 mu/ml) in triplicate for different lengths of time (12, 24, 48 h). After the exposure time, 10 μL of 5 mg/ml MTT solution was added to each well, and the cells were further incubated at 37 °C. After 4 h, the medium was discarded, and 100 μL DMSO was added to make dark blue formazan crystals at 37 °C for 10 min. The absorbance was recorded at 490 nm with an automatic microplate reader (Labsystems, Finland).

### Quantitative real-time PCR

TRIzol (TaKaRa Biotechnology, China) was used to extract total RNA for real-time fluorescence quantitative PCR according to the instructions. Using 500 ng total RNA as the material, cDNA was synthesized using reverse transcriptase (ReverTraAce reverse transcriptase, TaKaRa Biotechnology, China) and oligo dT primers (TaKaRa Biotechnology, China). The PCR primers were as follows:

Alp, Forward (F) 5′-CATCGCCTATCAGCTAATGCACA-3′,

Reverse (R) 5′-ATGAGGTCCAGGCCATCCAG-3′;

BMP2, F 5′-ACCGTGCTCAGCTTCCATCAC-3′,

R 5′-CTATTTCCCAAAGCTTCCTGCATTT-3′;

COL1, F 5′-GACATGTTCAGCTTTGTGGACCTC-3′,

R 5′-AGGGACCCTTAGGCCATTGTGTA-3′;

OSX, F 5′-CACCCATTGCCAGTAATCTTCGT-3′,

R 5′-GGACTGGAGCCATAGTGAGCTTCT-3′;

Runx2, F 5′-CATGGCCGGGAATGATGAG-3′,

R 5′-TGTGAAGACCGTTATGGTCAAAGTG-3′;

BGP, F 5′-GGACCCTCTCTCTGCTCACTCT-3′,

R 5′-CTTACTGCCCTCCTGCTTGG-3′;

beta-actin, F 5′-ACCCAGATCATGTTTGAGAC-3′,

R 5′-GTCAGGATCTTCATGAGGTAGT-3′.

Real-time PCR was performed on a LightCycler 480 (Roche Diagnostics, Germany). The initial denaturation step was 95 °C for 2 min, followed by 40 cycles of annealing at 95 °C for 10 s and 60 °C for 20 s. The Ct comparison method was used to calculate the mRNA expression level. We normalized the Ct value of the sample of interest to the Ct value of β-actin.

### Western blot analyses

Western blot analysis was used to extract cellular proteins in RIPA lysis buffer containing 1 mm PMSF. A BCA kit (Shenneng Bocai Biological Technology, China) was used to determine the protein concentration. Fifty micrograms of protein was electrophoresed on a 10% SDS-polyacrylamide gel under reducing conditions and then electrotransferred to a nitrocellulose membrane (Millipore). After blocking the membrane with 5% bovine serum albumin, the membrane was incubated with anti-Runx2 (1:600), anti-osterix (1:100) (Santa Cruz Biotechnology, USA) and anti-actin antibodies (1:3000), and then anti-rabbit or mouse secondary antibody (China ZSGB-Biotechnology) was used for detection. These images are detected with imaging equipment.

### Statistical analysis

All data are expressed as the mean ± SDs. Differences between groups were analyzed by one-way analysis of variance, and SPSS 17.0 software was used for the SNK test for mean comparisons. *P* < 0.05 was considered significant.

## Results

### Subject characteristics

The 114 men were divided into two groups based on their mean TSH levels. The low-level serum TSH group (TSH ≤ 1.65 mU/L) included 39 participants, and the high-level serum TSH group (TSH > 1.65 mU/L) included 75 participants. Table 1 presents the subject characteristics.

**Table 1:** Characteristics of the subjects in the two groups

	N	Age (y)	Height (cm)	Weight (kg)	BMI (kg/m^2^)
Low-level serum TSH group	39	44.46 ± 17.0	170.02 ± 6.45	68.48 ± 7.84	23.66 ± 2.11
High-level serum TSH group	75	40.81 ± 16.3	171.66 ± 6.50	70.19 ± 9.66	23.42 ± 2.91

The values are expressed as the means±SDs

*TSH* Thyroid-stimulating hormone, *BMI* Body mass index

### Interrelationships between serum TSH and BMD

The serum FT3 and FT4 levels between the two groups did not show significant changes as the serum TSH concentration increased. Forearm BMD was increased in the high-level serum TSH group compared with the low-level serum TSH group (*P* < 0.05) (Table 2).

**Table 2:** The forearm BMD, serum FT3 levels, and FT4 levels in the two groups

	N	FT3 (pmol/L)	FT4 (pmol/L)	BMD (g/cm^2^)
Low-level serum TSH group	39	5.6 ± 0.47	17.26 ± 2.0	0.48 ± 0.08
High-level serum TSH group	75	5.53 ± 0.47	17.28 ± 2.0	0.51 ± 0.06
*P*-value		0.632	0.818	0.018

The values are expressed as the means±SDs

*FT3* Free triiodothyronine, *FT4* Free tetraiodothyronine, *BMD* Bone mineral density

### Cell morphological identification of osteoblasts

**Fig. 1:**
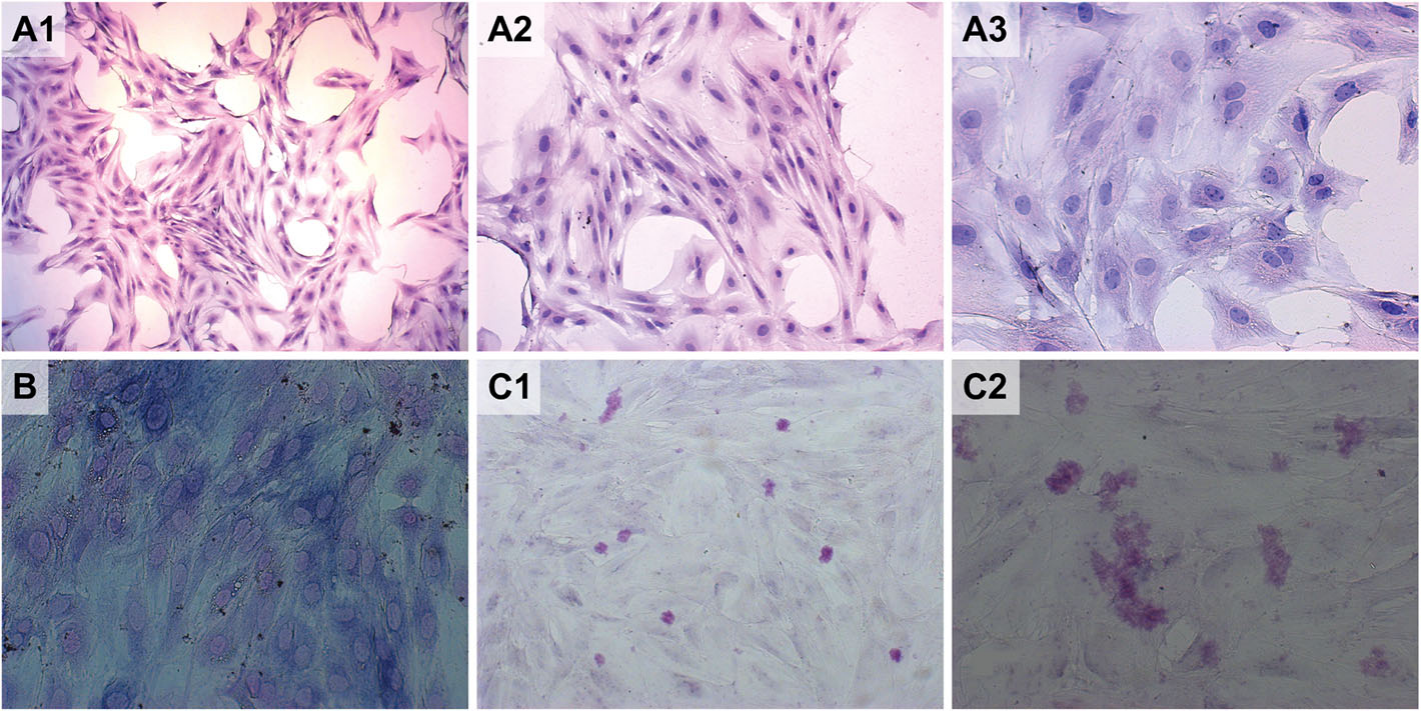
HE staining of P3 primary osteoblasts observed by microscopy. The cell surface had various protrusions, through which they were connected to adjacent cells. Large nuclei were located in the center or towards the side of the cells (A1*50, A2*100, A3*400). The cytoplasm appeared violet due to ALP staining (B*400). On d 16 of osteoblast culture, there were many round or elliptical opaque nodules that were stained red by Alizarin red (C1*100, C2*200)

HE staining was observed using a microscope (Fig. 1). The cell bodies were small and appeared triangular, square or polygonal, and some cells showed fusion. The cell surfaces had various protrusions through which they were connected to adjacent cells. Large nuclei were located in the center or near the side of the cells. Consistent with the typical biochemical characteristics of osteoblasts, the cytoplasm appeared violet. After 16 d of Alizarin red staining, many round or elliptical opaque red nodules were observed under a microscope. These findings confirmed that the nodules were mineralized nodules, further demonstrating that the cells had mineralization.

### Generation of the osteoblast growth curve

**Fig. 2:**
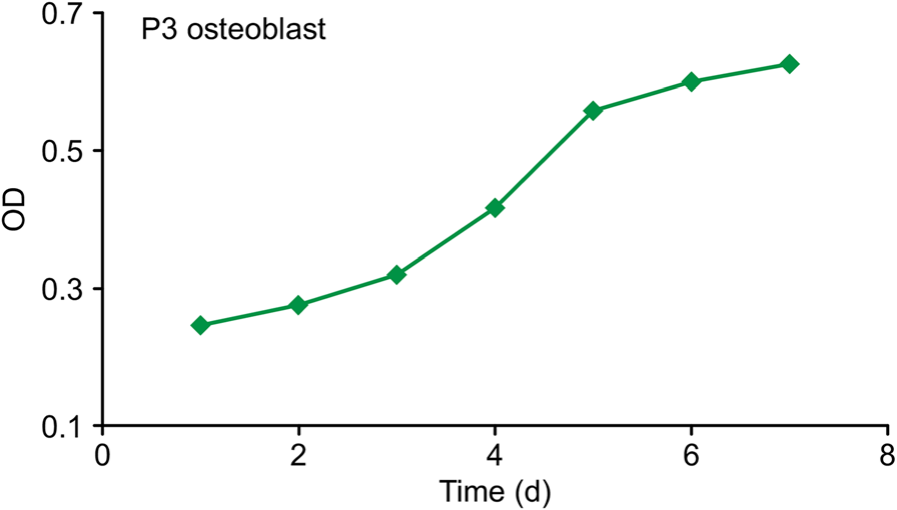
The growth trend of P3 primary osteoblasts is that of an S-shaped curve. The first 2 d after inoculation was considered the adjustment period; starting on d 3, cells entered the logarithmic phase, showed peak proliferation at approximately 5 d, and began to plateau on d 6. The growth trend of the osteoblasts was consistent with that in most of the literature reported

The growth trend of Sprague-Dawley (SD) rat osteoblasts was an S-shaped curve (Fig. 2). The first 2 d after inoculation was considered the adjustment period; at 3 d, the cells entered the logarithmic phase. Proliferation peaked at approximately 5 d and plateaued beginning at 6 d. The growth trend of osteoblasts was consistent with that reported in most of the literature.

### TSH promotes osteoblast proliferation

**Fig. 3:**
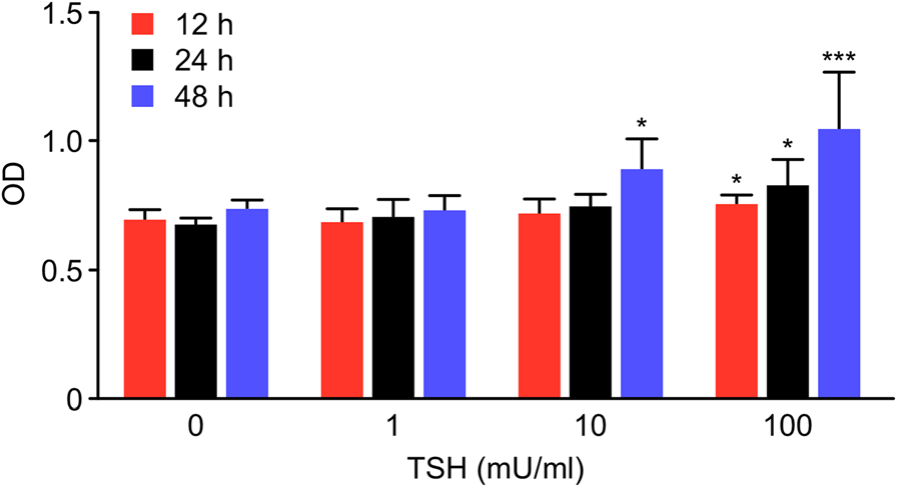
Cell proliferation is regulated by TSH in primary osteoblasts. Osteoblasts cocultured with different concentrations of TSH (0, 1, 10, and 100 mU/mL) were detected at 12, 24, and 48 h by MTT assays. The data are expressed as the means±SDs. *P < 0.05 versus the control group (TSH = 0 mU/mL) at the corresponding time period. ***P < 0.001 versus the control group (TSH = 0 mU/mL) at 48 h

We first investigated whether cell proliferation could be regulated by TSH in primary osteoblasts. Osteoblasts were treated with TSH for different lengths of time and different doses. MTT assays showed that at the 12 h and 24 h time points, the OD value of the TSH 100 mU/mL group was higher than that of the other three groups (*P* < 0.05), and the differences in the mean OD values between the other three groups were not significant (*P* > 0.05). After cultivation for 48 h, the mean OD value of the 10 mU/mL TSH group was higher than that of the control group (P < 0.05). The OD value of the TSH 100 mU/mL group was higher than that of the control group (*P* < 0.001), while there were no differences between the OD values of the 1 mU/mL experimental group and the control group (P > 0.05) (Fig. 3).

The results indicated that none of the above TSH concentrations had inhibitory effects on osteoblast proliferation. Treatment with 100 mU/mL showed the strongest promotion of cell proliferation. Furthermore, 10 mU/mL TSH promoted osteoblast proliferation, but the effect was weaker than that of the 100 mU/mL treatment. Treatment with 1 mU/mL had no effect on osteoblast proliferation. In conclusion, TSH can promote osteoblast proliferation.

### TSH promotes osteoblast differentiation

**Fig. 4:**
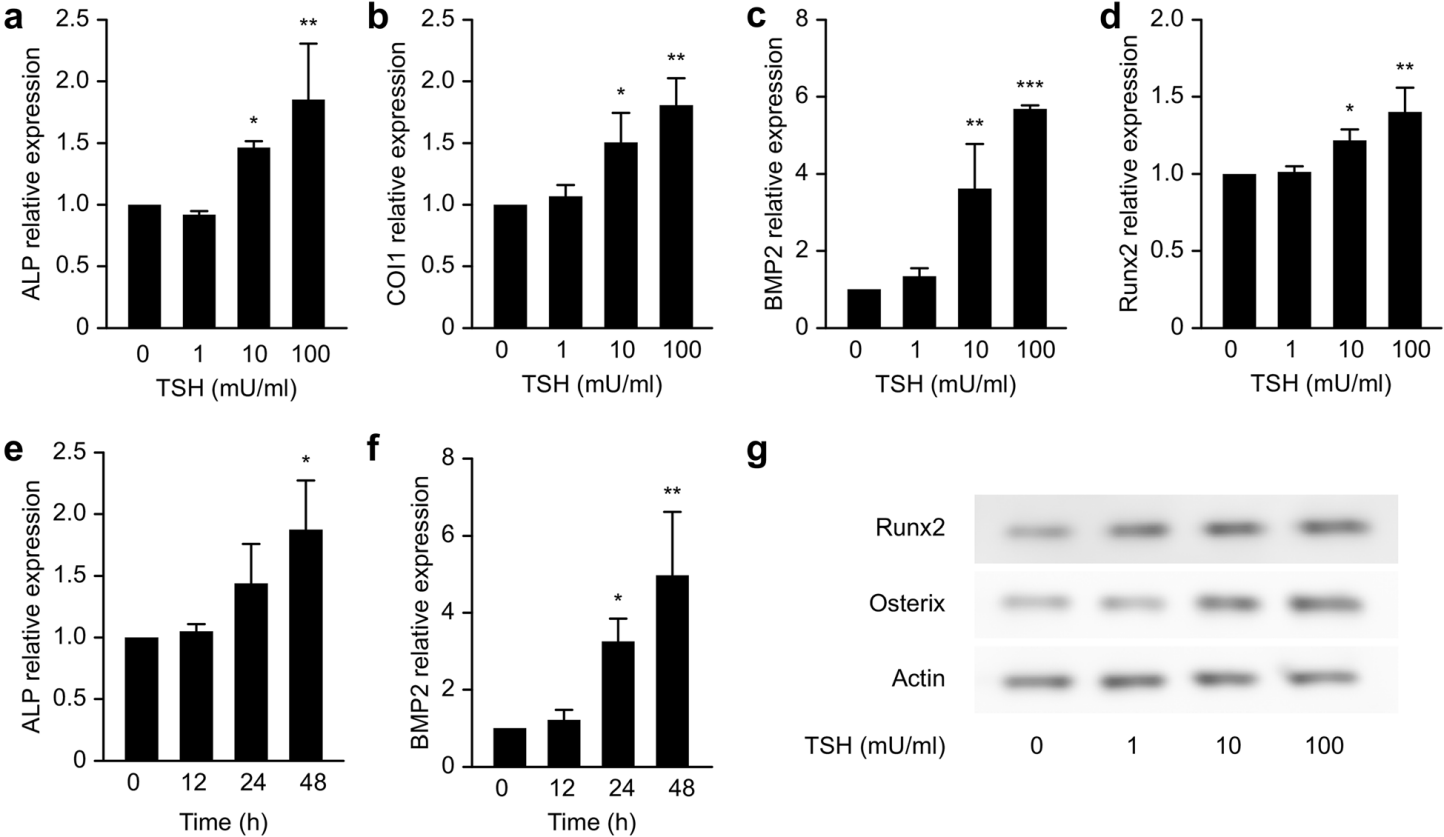
Effect of TSH on the mRNA expression of ALP, COI1, BMP2 and Runx2. The mRNA expression of ALP (**a**), COI1 (**b**), BMP2 (**c**) and Runx2 (**d**) was determined by real-time RT-PCR. ALP (**e**) and BMP2 (**f**) mRNA levels were detected at 0, 12, 24 and 48 h of 100 mU/mL TSH treatment. The data are expressed as the means±SDs. *P < 0.05, **P < 0.01, ***P < 0.001 versus the control group. As shown by western blot analysis, TSH (10 and 100 mU/mL) promoted Runx2 and osterix protein expression (**g**)

After starving for 2 h, primary osteoblasts were cultured in the absence or presence of TSH (1, 10 and 100 mU/mL) for 48 h. Osteoblast differentiation was analyzed by determining the RNA levels of the osteoblast marker proteins ALP, COL1, bone morphogenetic protein (BMP)2 and Runx2. As shown in Fig. 4, TSH at concentrations of 10 mU/mL and 100 mU/mL significantly increased the mRNA levels of ALP, COL1 and Runx2 compared with those of the control (*P* < 0.05, *P* < 0.01), while the differences between the 1 mU/mL TSH treatment group and the control group were not significant (*P* > 0.05). BMP2 mRNA levels in the groups treated with 10 and 100 mU/mL TSH were significantly increased compared with those in the control group (*P* < 0.01, *P* < 0.001), while there was no significant difference between the 1 mU/mL TSH treatment group and the control group (P > 0.05). All of the above results showed that TSH could increase the mRNA levels of genes related to osteoblast function, such as ALP, COL1, BMP2 and Runx2, in a dose-dependent manner. We also detected the mRNA levels of ALP and BMP2 at the 12, 24, and 48 h time points after treatment with 100 mU/mL TSH. ALP and BMP2 activity increased over time and peaked at 48 h (*P* < 0.05, P < 0.01). Moreover, the effects of TSH on Runx2 and osterix protein expression were analyzed by western blot analysis. As shown in Fig. 4(g), TSH (10 and 100 mU/mL) promoted Runx2 and osterix protein expression.

## Discussion

Studies have reported associations between higher than normal levels of FT3 and FT4 with reduced bone density and an increased risk of nonvertebral fractures. High FT3 and FT4 levels are associated with low bone density in the hip joint, and high FT4 levels are also associated with potential bone loss at the hip joint [25]. However, thyroid hormone has been shown to stimulate cells to secrete insulin-like growth factor I, which further stimulates osteoblast progenitor cells and ultimately promotes the differentiation and proliferation of osteoblasts. T3 binds to thyroid hormone receptors on osteoblasts, affecting cell proliferation, protein synthesis, and matrix formation, but high-dose T3 inhibits osteoblast proliferation. T3 binds to thyroid hormone receptors on osteoclasts and promotes the proliferation and activity of these cells. Based on these results, thyroid hormone exerts a regulatory effect on bone growth, maturation and transformation [26].

As a result, researchers have been unable to exclude a role for T3 in patients with hyperthyroidism and have only studied the role of TSH in osteoporosis. Subclinical thyroid disease refers to a normal serum thyroid concentration, with only the TSH concentration being higher or lower than the normal range. Therefore, this situation is considered an appropriate model for studying the direct effects of TSH on bone metabolism while eliminating the direct effects of thyroid hormone on bone metabolism [27]. Of the 5458 individuals in the six prospective cohorts (mean age: 72 years, 49.1% female), 451 (8.3%) were diagnosed with subclinical hypothyroidism, and 284 (5.2%) were diagnosed with SH. At 36,569 follow-up years, patients with SH had greater annual bone loss in the femoral neck than patients with hyperthyroidism. SH is associated with increased bone loss at the femoral neck, which may increase the risk of fracture [28]. In older women without significant thyroid dysfunction [29], low TSH levels are independently associated with decreased bone density in the femoral neck. A meta-analysis of 1371 studies and 70,298 participants also showed that subclinical thyroid dysfunction is an important risk factor for fractures [30].

Suppressed levels of TSH correlate with low BMD in hyperthyroidism [31], particularly in postmenopausal women [32]. Even TSH levels in the normal range show a similar relationship in the elderly and are associated with an increased risk of hip fracture in healthy women [33]. Thus, the duration of TSH inhibition is a predictor of major osteoporotic fractures. A cross-sectional study reported a high incidence of vertebral fractures during TSH inhibition, even in patients with a normal bone density. Vertebral fractures are very common in women with low TSH levels and bone density in the osteoporotic range [34]. Siderova et al. described the negative effect of hyperthyroidism on BMD. In contrast, TSH exerts a positive effect on BMD. TSH receptor antibodies are often present at high titers in patients with Graves’ ophthalmopathy, and they may exert a protective effect on bone [35].

In the present study, after adjustment for age, body mass index, and serum levels of FT4, higher serum TSH levels were observed in the group with a higher BMD than in the low serum TSH group, and normal thyroid function was observed in the male group. Furthermore, normal thyroid function was observed in the male group (*P* < 0.05), indicating that the BMD of males also increases with increased serum TSH concentration. These results are consistent with previous studies.

Epigenetic mesenchymal osteoblasts derived from undifferentiated pluripotent stem cells synthesize and secrete osteoid components, such as collagen and glycoproteins, which participate in osteoid calcification and regulate osteoclast activity. These molecules play an important role in bone formation. Primary osteoblasts are often isolated as the main cell source for in vitro experiments. Two main methods of osteoblast isolation have been described: enzymatic digestion and tissue blocking. The former is more widely used than the latter. Osteoblasts were successfully extracted from rat skulls by enzymatic digestion. The cells grew well in culture and showed activity. Because fibroblasts exhibit high and rapid adhesion, we used the repeated adhesion method to remove fibroblasts and obtain high-purity osteoblasts. The cells were identified by HE staining and morphological observations of biological characteristics, and the extracted cells were confirmed to have typical features of osteoblasts.

MTT colorimetry showed that 10 mU/mL and 100 mU/mL TSH promoted the proliferation of osteoblasts, and the dose of 100 mU/mL was the most effective. Using real-time PCR analysis, genes related to osteoblast function, such as COL1, BMP2, and Runx2, were further detected in cells treated with different concentrations of TSH. In this study, TSH increased the expression of genes related to osteoblast function, such as ALP, COL1, BMP2 and Runx2, compared with the control group. At concentrations of 10 mU/mL and 100 mU/mL, the changes in gene expression were significant (*P* < 0.05, *P* < 0.01, and *P* < 0.001). The effect of 100 mU/mL TSH on the mRNA levels of genes associated with osteoblast function, including ALP, COL1, BMP2 and Runx2, was stronger than the effect of 10 mU/mL TSH, suggesting that TSH exerts a dose-dependent effect on the mRNA levels of genes related to osteoblast function. Then, we investigated the mRNA expression of ALP and BMP2 at 12, 24, and 48 h. TSH increased the mRNA levels of ALP and BMP2 over time, confirming that TSH directly promotes osteoblast differentiation and bone formation.

BMPs are generally considered to be the main factors that induce bone repair. According to mechanistic analysis, BMPs bind to BMPR, promoting its association with Smad coactivators and p38-MAPK, thereby activating the downstream transcription factor Runx2. This transcription factor directly targets the promoter regions of ALP and osteocalcin to regulate osteogenesis and differentiation [36]. The Runx2 protein regulates osteoblast differentiation by directly affecting the transcriptional activity of ALP, BGP and the downstream COL1 and OPN molecules [37]. BMP2 is involved in TSH-induced osteoblast proliferation and differentiation. Therefore, we speculated that the BMP-Smads-Runx2-osterix pathway might be involved in this process. Further studies are needed to explore the specific mechanisms.

TSHR mediates signaling through many different pathways, although the GS-cAMP pathway is considered the primary pathway. The development of functionally biased TSHR agonists, in which one signaling pathway may be superior to the other, may facilitate the development of drugs that selectively modulate treatment-related physiological functions, such as PTH1R signaling [38]. As shown in the study by Boutin, TSH-mediated upregulation of IL-11, ALPL, and OPN occurs through different G protein-coupled signaling pathways. Based on these findings, the development of TSHR agonists biased towards the ashutin-ashutin-1 and Gq/11-ERk1/2 pathways may contribute to the treatment of osteoporosis [39].

In recent years, small molecules have received increasing attention as therapeutic options for regulating TSHR signaling [40]. Their chemical properties make them resistant to proteolytic enzymes, making these molecules ideal therapeutic agents [41]. Molecular docking and experimental studies have shown that the TSH protein binds to TSHR and signals through TSHR [42, 43]. In addition to macrophages, the mouse pituitary is also a source of a new TSH splice variant (TSH-KVV), which may retain its biological effects [42–44]. A small molecule, MS-438, appears to increase osteoblast formation through the PKA signaling pathway [45]. Other studies have reported the biological effects of small molecules on osteoblasts overexpressing TSHR [46], and two small-molecule TSHR antagonists with a lower binding affinity than required for clinical use have been identified [47, 48].

We only conducted cell-based experiments, and our clinical examination was a retrospective study of a small number of patients. Because a single bone density measurement in the forearm does not adequately reflect bone metabolism, further studies, such as in vivo animal experiments and large-sample clinical studies, are needed to accurately assess signal transduction in osteoblasts. Studies aiming to elucidate the relationship between TSH and osteoporosis will provide a new basis for the treatment of thyroid disease and osteoporosis.

## Conclusion

In the present study, we demonstrated that TSH serum concentrations positively correlated with BMD in men with normal thyroid function. TSH can promote osteoblast proliferation. In addition, we confirmed that TSH increased the expression of key osteoblast differentiation genes, such as ALP, BMP2, COL1, and Runx2, in a dose-dependent manner. TSH directly or indirectly affects the osteoblast microenvironment and promotes bone differentiation and bone formation.

## Supplementary Information

**Additional file 1.**

## Data Availability

The datasets used in the analyses described in this study are available from the corresponding author on reasonable request.

## Abbreviations

TSH: Thyroid-stimulating hormone
FT3: Free triiodothyronine
FT4: Free thyroxine
BMD: Bone mineral density
TSHR: Thyroid-stimulating hormone receptor
ES: Embryonic stem
BMI: Body mass index
DXA: Dual-energy X-ray absorptiometry
ALP: Alkaline phosphatase
DMSO: Dimethyl sulfoxide
BMPs: Bone morphogenetic proteins

## References

[1] Martin T, Gooi JH (2009). Molecular mechanisms in coupling of bone formation to resorption. Crit Rev Eukaryot Gene Expr.

[2] Matsuo K (2008). Osteoclast-osteoblast communication. Arch Biochem Biophys.

[3] Phan TC, Xu J (2004). Interaction between osteoblast and osteoclast: impact in bone disease. Histol Histopathol.

[4] Zhang W, Zhang Y, Liu Y (2014). Thyroid-stimulating hormone maintains bone mass and strength by suppressing osteoclast differentiation. J Biomech.

[5] Bassett JH, Williams GR (2016). Role of thyroid hormones in skeletal development and bone maintenance. Endocr Rev.

[6] Williams GR, Bassett JHD (2018). Thyroid diseases and bone health. J Endocrinol Investig.

[7] Biondi B, Cooper DS (2010). Benefits of thyrotropin suppression versus the risks of adverse effects in differentiated thyroid cancer. Thyroid..

[8] Delitala AP, Scuteri A, Doria C (2020). Thyroid Hormone Diseases and Osteoporosis. J Clin Med.

[9] Xu N, Wang Y, Xu Y (2020). Effect of subclinical hyperthyroidism on osteoporosis: a meta-analysis of cohort studies. Endocrine..

[10] Faber J, Galløe AM (1994). Changes in bone mass during prolonged subclinical hyperthyroidism due to L-thyroxine treatment: a meta-analysis. Eur J Endocrinol.

[11] Kung AW, Yeung SS (1996). Prevention of bone loss induced by thyroxine suppressive therapy in postmenopausal women: the effect of calcium and calcitonin. J Clin Endocrinol Metab.

[12] Müller CG, Bayley TA, Harrison JE, Tsang R (1995). Possible limited bone loss with suppressive thyroxine therapy is unlikely to have clinical relevance. Thyroid..

[13] Guo CY, Weetman AP, Eastell R (1997). Longitudinal changes of bone mineral density and bone turnover in postmenopausal women on thyroxine. Clin Endocrinol.

[14] Mendonça Monteiro de Barros G, Madeira M, Vieira Neto L (2016). Bone mineral density and bone microarchitecture after long-term suppressive levothyroxine treatment of differentiated thyroid carcinoma in young adult patients. J Bone Miner Metab.

[15] Wojcicka A, Bassett JH, Williams GR (2012). Mechanisms of action of thyroid hormones in the skeleton. Biochim Biophys Acta.

[16] Bassett JH, van der Spek A, Logan JG (2015). Thyrostimulin regulates Osteoblastic bone formation during early skeletal development. Endocrinology.

[17] Abe E, Marians RC, Yu W (2003). TSH is a negative regulator of skeletal remodeling. Cell.

[18] Bassett JH, Williams AJ, Murphy E (2008). A lack of thyroid hormones rather than excess thyrotropin causes abnormal skeletal development in hypothyroidism. Mol Endocrinol.

[19] Sampath TK, Simic P, Sendak R (2007). Thyroid-stimulating hormone restores bone volume, microarchitecture, and strength in aged ovariectomized rats. J Bone Miner Res.

[20] Hase H, Ando T, Eldeiry L (2006). TNFalpha mediates the skeletal effects of thyroid-stimulating hormone. Proc Natl Acad Sci U S A.

[21] Gogakos AI, Duncan Bassett JH (2010). Thyroid and bone. Arch Biochem Biophys.

[22] Baliram R, Latif R, Berkowitz J (2011). Thyroid-stimulating hormone induces a Wnt-dependent, feed-forward loop for osteoblastogenesis in embryonic stem cell cultures. Proc Natl Acad Sci U S A.

[23] Tsai JA, Janson A, Bucht E, Kindmark H, Marcus C, Stark A, Rawet Zemack H, Torring O. Weak evidence of thyrotropin receptors in primary cultures of human osteoblast-like cells. Calcif Tissue Int. 2004;74(5):486–91. 10.1007/s00223-003-0108-3. 10.1007/s00223-003-0108-3 14961213

[24] Wang J, Zhang W, Yu C, Zhang X, Zhang H, Guan Q, Zhao J, Xu J (2015). Follicle-stimulating hormone increases the risk of postmenopausal osteoporosis by stimulating osteoclast differentiation. PLoS One.

[25] Gogakos A, Logan JG, Waung JA (2014). THRA and DIO2 mutations are unlikely to be a common cause of increased bone mineral density in euthyroid post-menopausal women. Eur J Endocrinol.

[26] Allain TJ (1993). Thyroid hormones and bone. J Endocrinol.

[27] Lee WY, Oh KW, Rhee EJ (2006). Relationship between subclinical thyroid dysfunction and femoral neck bone mineral density in women. Arch Med Res.

[28] Segna D, Bauer DC, Feller M (2018). Association between subclinical thyroid dysfunction and change in bone mineral density in prospective cohorts. J Intern Med.

[29] Ding B, Zhang Y, Li Q (2016). Low thyroid stimulating hormone levels are associated with low bone mineral density in femoral neck in elderly women. Arch Med Res.

[30] Blum MR, Bauer DC, Collet TH (2015). Subclinical thyroid dysfunction and fracture risk: a meta-analysis. JAMA.

[31] Svare A, Nilsen TI, Bjøro T (2009). Hyperthyroid levels of TSH correlate with low bone mineral density: the HUNT 2 study. Eur J Endocrinol.

[32] Kim MK, Yun KJ, Kim MH (2015). The effects of thyrotropin-suppressing therapy on bone metabolism in patients with well-differentiated thyroid carcinoma. Bone.

[33] Leader A, Ayzenfeld RH, Lishner M (2014). Thyrotropin levels within the lower normal range are associated with an increased risk of hip fractures in euthyroid women, but not men, over the age of 65 years. J Clin Endocrinol Metab.

[34] Mazziotti G, Formenti AM, Frara S (2018). High prevalence of radiological vertebral fractures in women on thyroid-stimulating hormone-suppressive therapy for thyroid carcinoma. J Clin Endocrinol Metab.

[35] Siderova M, Hristozov K (2018). TSH-receptor antibodies may prevent bone loss in pre- and postmenopausal women with Graves’ disease and Graves’ orbitopathy. Arch Endocrinol Metab.

[36] Jeon EJ, Lee KY, Choi NS (2006). Bone morphogenetic protein-2 stimulates Runx2 acetylation. J Biol Chem.

[37] Matsubara T, Kida K, Yamaguchi A (2008). BMP2 regulates Osterix through Msx2 and Runx2 during osteoblast differentiation. J Biol Chem.

[38] Sivertsen B, Holliday N, Madsen AN (2013). Functionally biased signalling properties of 7TM receptors - opportunities for drug development for the ghrelin receptor. Br J Pharmacol.

[39] Boutin A, Neumann S (2016). Multiple Transduction Pathways Mediate Thyrotropin Receptor signaling in Preosteoblast-Like Cells. Endocrinology.

[40] Davies TF (2015). Targeting the thyroid-stimulating hormone receptor with small molecule ligands and antibodies. Expert Opin Ther Targets.

[41] Baliram R, Latif R, Zaidi M (2017). Expanding the role of thyroid-stimulating hormone in skeletal physiology. Front Endocrinol (Lausanne).

[42] Baliram R, Chow A, Huber AK (2013). Thyroid and bone: macrophage-derived TSH-β splice variant increases murine osteoblastogenesis. Endocrinology.

[43] Baliram R, Latif R, Morshed SA (2016). T3 regulates a human macrophage-derived TSH-β splice variant: implications for human bone biology. Endocrinology.

[44] Vincent BH, Montufar-Solis D, Teng BB (2009). Bone marrow cells produce a novel TSHbeta splice variant that is upregulated in the thyroid following systemic virus infection. Genes Immun.

[45] Latif R, Ali MR, Ma R (2015). New small molecule agonists to the thyrotropin receptor. Thyroid.

[46] Boutin A, Eliseeva E, Gershengorn MC (2014). β-Arrestin-1 mediates thyrotropin-enhanced osteoblast differentiation. FASEB J.

[47] Neumann S, Huang W, Titus S (2009). Small-molecule agonists for the thyrotropin receptor stimulate thyroid function in human thyrocytes and mice. Proc Natl Acad Sci U S A.

[48] Latif R, Realubit RB, Karan C (2016). TSH receptor signaling abrogation by a novel small molecule. Front Endocrinol (Lausanne).

