# Supplementary material for: Thyroid-stimulating hormone decreases the risk of osteoporosis by regulating osteoblast proliferation and differentiation
Source: BMC Endocr Disord. 2021 Mar 16;21:49. doi: 10.1186/s12902-021-00715-8 (PMC7968288; doi:10.1186/s12902-021-00715-8)
Supplement: Supplementary file 1 — Additional file 1. [file 12902_2021_715_MOESM1_ESM.docx]

[Respondent Number Time](javascript:;)

[Investigator's Name Final Diagnosis](javascript:;)

**Questionnaire**

Epidemiological investigation of thyroid disease and osteoporosis in the community of Jinan, China. **General:**

1. Name:

2. Sex: 1-Male, 2-Female

If it is a woman, continue to ask: menopause? 1-Yes, 2-No

3. Date of birth: Age:

4. Home address:

5. ID number:

Work unit:

6. Telephone:

7. Primary contact: name: telephone:

8. Ethnicity:

9. Educational level:

1-primary school, 2-junior high school, 3-senior high school,

4-university/college, 5-postgraduate, 6-not enrolled.

10. Occupation:

11. Average income per month:

1-<1000 yuan, 2-1000-2000 yuan, 3-2000-3000 yuan,

4-3000-4000 yuan, 5-5000-10000 yuan, 6-10,000-30,000 yuan,

7-30,000 -50,000 yuan, 8-50,000-100,000 yuan, 9->100,000 yuan

12. Health insurance status:

1-rural cooperative medical care, 2-public expense, 3-out-of-pocket payments, 4-health insurance, 5-commercial insurance, 6-others

13. If the respondent is a woman, please ask the following questions about her menstrual and reproductive history:

Reproductive history: 1-abortion, 2-stillbirth, 3-neonatal death, 4-normal

Menstrual period: menarche ( years old); last menstrual period:

Numbers of pregnancies: live births: stillbirths:  abortions:

First pregnancy: ( years old)

Last pregnancy: ( years old)

Breastfeeding: ( months)

**Iodine:**

1. Salt source: 1-commercially available iodized salt 2-no iodized salt 2. Did you eat seaweed and nori yesterday? 1-Yes 2-No

3. Frequency of eating seaweed or nori: 1-often (eat weekly), 2-occasionally (eat every month) 3-do not eat

4. Take iodine-containing drugs: 1-Yes 2-No

If yes, continue to ask what kind of medicine:

1-acetylamide iodized furanone 2-potassium iodide 3- kumb 4-stone medicine 5-iodine-containing vitamins 6-Huaxin tablets 7-seaweed 8-others

Time the respondent starting taking that medicine:

Last time the respondent took that medicine:

Daily dosage:

**History of medication:**

|  | The name of the drug | Dose | Start date | Duration (months) |
| --- | --- | --- | --- | --- |
| Calcium |  |  |  |  |
| Vitamins |  |  |  |  |
| Anti-osteoporosis drugs |  |  |  |  |
| Diuretics |  |  |  |  |
| Liping or statins |  |  |  |  |
| Metformin |  |  |  |  |
| Insulin |  |  |  |  |
| Thyroxine tablets or Yujiale |  |  |  |  |

**History of disease:**

1. History of thyroid disease:

Previous clinical hyperthyroidism: clear diagnosis time, hospitalization, treatment (drugs, radiotherapy, surgery), oral drugs used, start of medication, daily dose, medication duration, cessation of medication; radiation dose and frequency; surgical operation; current symptoms; recent test results.      
Previous clinical hypothyroidism: clear diagnosis time, hospitalization, drug treatment (oral drugs used, start of medication, daily dose, medication duration, medication cessation).

[Thyroid](file:///C:\Program%20Files\Youdao\Dict\7.2.0.0511\resultui\dict\?keyword=thyroid) [nodule](file:///C:\Program%20Files\Youdao\Dict\7.2.0.0511\resultui\dict\?keyword=nodule): clear treatment of thyroid nodules: e.g. diagnosis (thyroid nodules, thyroid tumors), diagnosis date, medication

history, surgery history; diagnosis date and pathologic type of

thyroid cancer (emaciation, mass enlargement).

1. Other diseases.

1-Yes 2-None

If yes, please ask:

Endocrine conditions: 1-diabetes 2-hyperthyroidism

3-hyperparathyroidism 4-galaetorrhea-amenorrhea

If the respondent has diabetes, please ask:

1.1 How long ago was the diabetes diagnosed?

1-<=3 months 2- 4-6 months 3-7-12 months 4-1-2 years 5-2-3 years 5->=3 years.

1.2 What type of diabetes does the respondent have? 1-Type 1 2-Type 2 3-Other (please indicate).

Serious organ disease:

1-chronic liver disease 2-chronic kidney disease 3-heart disease 4-cerebrovascular disease 5-tumor.

3. Other: 1-blood system disease 2-rheumatoid arthritis 3-others. **History of surgery:**

1-Yes 2-None...

If yes, please ask:

1-history of gastrointestinal resection 2-hysterectomy 3-unilateral oophorectomy 4-bilateral oophorectomy 5-other...

Age at the time of the surgery and cause:

**Family history:**

(i) Thyroid disease: 1-Yes 2-No.

If yes, please ask:

Relationship:

Disease:

1-hyperthyroidism 2-[hypothyroidism](file:///C:\Program%20Files\Youdao\Dict\7.2.0.0511\resultui\dict\?keyword=hypothyroidism) 3-goiter 4-[thyroid](file:///C:\Program%20Files\Youdao\Dict\7.2.0.0511\resultui\dict\?keyword=thyroid) [nodule](file:///C:\Program%20Files\Youdao\Dict\7.2.0.0511\resultui\dict\?keyword=nodule) 5-thyroid cancer 6-thyroid tumor 7-other.

(ii) Diabetes: 1-Yes 2-No.

If yes, please ask:

Relationship:

Disease:

1-Type 1 2-Type 2 3-other (if selected, please specify the type).

(iii) Obesity: 1-Yes 2-No.

If yes, please ask:

Relationship:

(iv) Blood lipid abnormalities: 1-Yes 2-no.

If yes, please ask:

Relationship:

(v) Heart and cerebrovascular diseases: 1-Yes 2-No.

If yes, please ask:

Relationship:

Disease:

1-coronary heart disease 2-arteriosclerosis 3-hypertension

4-cerebral infarction 5-cerebral hemorrhage

(vi) Osteoporosis: 1-Yes 2-No.

If yes, please ask:

Relationship:

Age at occurrence: years old

(vii) Fracture: 1-Yes 2-No.

If yes, please ask:

Relationship:

Age at occurrence: years old

Fracture site:

Cause of fracture:

This is the end of the questionnaire; thank you for your cooperation!
